# BinTree Seeking: A Novel Approach to Mine Both Bi-Sparse and Cohesive Modules in Protein Interaction Networks

**DOI:** 10.1371/journal.pone.0027646

**Published:** 2011-11-28

**Authors:** Qing-Ju Jiao, Yan-Kai Zhang, Lu-Ning Li, Hong-Bin Shen

**Affiliations:** 1 Department of Automation, Shanghai Jiao Tong University, and Key Laboratory of System Control and Information Processing, Ministry of Education of China, Shanghai, China; 2 Department of Physics, Shanghai Jiao Tong University, Shanghai, China; Semmelweis University, Hungary

## Abstract

Modern science of networks has brought significant advances to our understanding of complex systems biology. As a representative model of systems biology, Protein Interaction Networks (PINs) are characterized by a remarkable modular structures, reflecting functional associations between their components. Many methods were proposed to capture cohesive modules so that there is a higher density of edges within modules than those across them. Recent studies reveal that cohesively interacting modules of proteins is not a universal organizing principle in PINs, which has opened up new avenues for revisiting functional modules in PINs. In this paper, functional clusters in PINs are found to be able to form unorthodox structures defined as bi-sparse module. In contrast to the traditional cohesive module, the nodes in the bi-sparse module are sparsely connected internally and densely connected with other bi-sparse or cohesive modules. We present a novel protocol called the BinTree Seeking (BTS) for mining both bi-sparse and cohesive modules in PINs based on Edge Density of Module (EDM) and matrix theory. BTS detects modules by depicting links and nodes rather than nodes alone and its derivation procedure is totally performed on adjacency matrix of networks. The number of modules in a PIN can be automatically determined in the proposed BTS approach. BTS is tested on three real PINs and the results demonstrate that functional modules in PINs are not dominantly cohesive but can be sparse. BTS software and the supporting information are available at: www.csbio.sjtu.edu.cn/bioinf/BTS/.

## Introduction

Most biological characteristics arise from complex interactions between the cell's numerous constituents, such as proteins, DNA, RNA, and small molecules [1]–[5]. Therefore, a great challenge in systems biology is to understand the structure and the dynamics of the complex intercellular networks of interactions that contribute to the structure and function of a living cell [5]. Biological functions seldom rely on individual proteins to perform particular cellular tasks; quite on the contrary, they are generally discovered from interactions among multiple members to form highly-organized modules, where proteins often interact intimately and intensively [6]. Modules are of interest because they often correspond to functional subunits [5], such as protein complexes [6], [7] or social spheres [8]. Revealing these modular constituents in networks will undoubtedly bring richer biological information in gaining insights into dynamic of molecular systems on a new landscape. As a representative example in complex biological systems, PIN is widely used to predict protein functions [9]–[11] because its dynamic and modular structures are considered to be capable of providing more significant and direct evidences in formation of protein functions. One of the examples is known as the automatic protein complex prediction method, where protein complexes generally correspond to clusters in a PIN because proteins in a complex are strongly interactive with each other [12]. Considering the importance of the module information buried in a PIN, a number of mathematical and computer algorithms have been proposed to tackle module and protein complex detections in protein interaction networks [6], [13]–[18].

**Figure 1:**
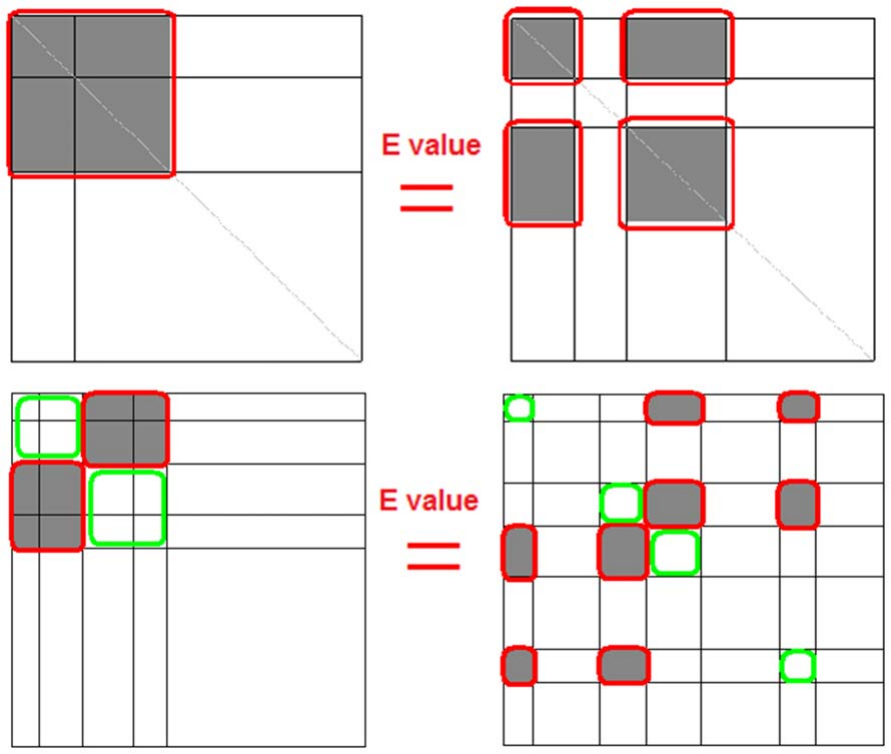
An example of the over-split results for diagonal and non-diagonal modules. The over-split issue in the Pinkert method means that the error function E value does not change if a big diagonal or non-diagonal module is split two or multi modules.

However, it has been revealed that the cohesive modules did not completely depict various functional units in PINs. In 2007, Wang *et al* analyzed the yeast PINs including PIC network that includes protein complex data and PEC network that excludes all edges inferred from protein complexes, and they found that the identified modules lack obvious correspondence to functional units [19]. In 2010, Pinkert *et al* presented an alternative approach different from prior definitions of what actually constitutes a “module” to detect functional modules in PINs. They applied the method (denoted as Pinkert method in the following section) to the PIN from the Human Protein Reference Database (HPRD) and found some self-linking and isolated nodes that were proved to be functional modules [20]. What's more, the authors found some significant non-diagonal modules, which were functionally related and can provide better description for the characteristics of a protein interaction network than cohesive modules alone. Therefore, the common notion that cohesive module is considered as the sole organizing structure for functional unit is challenged. A Simulated Annealing (SA) based algorithm was also proposed in [20] for the purpose of finding both cohesive and sparse modules. Although this method was demonstrated effective, it is highly dependent on the parameters chosen for optimization in SA, for example, initial temperature and cooling factor, where the most difficult parameter could be the number of modules in the network should be predefined. By setting different number of clusters, one can get totally different outputs. This parameter is particularly hard to be set properly when the network size is large. Another disadvantage of optimizing modularity E-value by SA [20] is for diagonal and non-diagonal modules, the over-split phenomena can't be avoided in the whole process (Figure 1).

Unlike previous approaches that extract clusters or modules by identifying groups of proteins with similar patterns of interaction to other proteins, this paper focuses on an unorthodox structure of module that is defined as bi-sparse module. The members in bi-sparse module are sparsely connected internally and densely connected with other bi-sparse or cohesive modules. Accordingly, we proposed a BinTree Seeking (BTS) method based on the Edge Density of Module (EDM) and binary tree theory to mine both bi-sparse and cohesive functional modules. Different from the existing literatures, which focus on grouping nodes [21] or optimizing modularity [16], [22]–[24] in networks, the new BTS method takes full advantage of the relationship between network edges and nodes and binary search tree theory. Another merit of BTS approach is that it does not need to set the number of modules beforehand and this important parameter can be automatically identified in BTS based on a given evaluation criterion. By applying the BTS method to analyze the protein Kinase and Phosphatase Interaction Network (KPIN) [25], a human protein interaction network from the I2D database [26], and a yeast interaction network from DIP database [27], we finally obtain functional clusters composed of both cohesive and bi-sparse modules.

## Results

### The results by applying BTS on synthetic network

Detection of blocks is a classic issue in complex network studies and many methods were proposed in the literature [28]–[30]. The outputs from traditional approaches are dominantly cohesive clusters in the objective network, which are considered functional important. As a significant complement, it has been revealed recently that sparse module also could be important functional units although the links among their members are very sparse [20].

**Figure 2:**
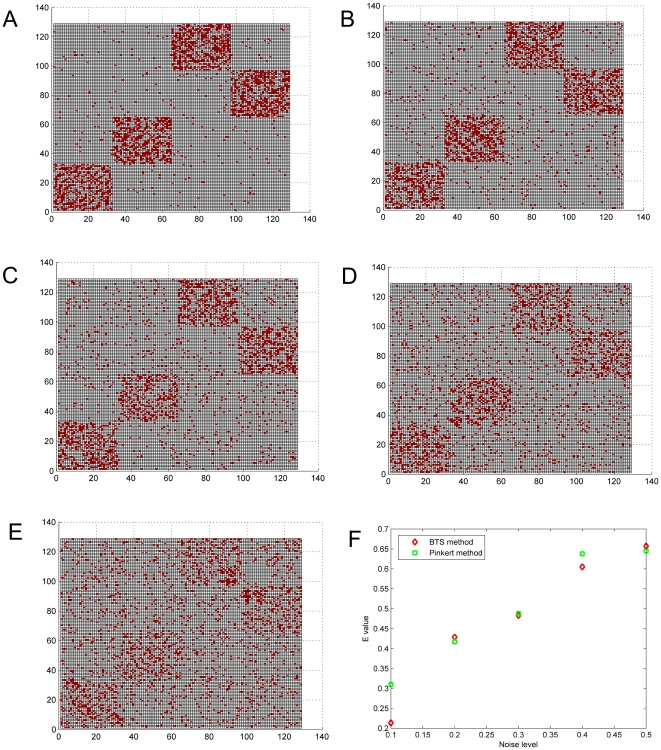
The E values for the two methods on 5 complex networks. Synthetic networks composed of 2 cohesive clusters and 2 bi-partite structures: (A) with 10% noise; (B) with 20% noise; (C) with 30% noise; (D) with 40% noise; (E) with 50% noise; and (F) E-value comparison results between Pinkert method and proposed BTS.

A synthetic benchmark network that is composed of 128 nodes was constructed consisting of four modules, two of which are cohesive clusters and the other two form bi-partite structures. In order to effectively demonstrate the robustness of the proposed BTS method, 5 noisy complex networks with noise level of 0.1∼0.5 were constructed by adding noise to the original benchmark data (Figure 2(A)–(E)), where the way to add noise is the same as described in the Pinkert method [20]. The proposed BTS and the Pinkert method (the number of classes is set to be 4) were both employed to mine the clusters in these 5 noisy networks. Figure 2(F) compares the E-value results from the two methods respectively. From the results we can find that BTS can get smaller E-values on 3 tested networks of noise level equal to 0.1, 0.3, and 0.4; Pinkert method performs better on the other two networks. Our experiments also show that E-values in the Pinkert method can be changed dramatically when the number of classes is set to other values.

### The results of applying BTS to real PINs data

By using the proposed BTS method, we analyzed the KPI protein interaction network, DIP yeast protein interaction network, and BIND human protein interaction network. As a result, we get 29, 59, and 65 modules respectively on these three PINs (see Figure S1 for details).

**Figure 3:**
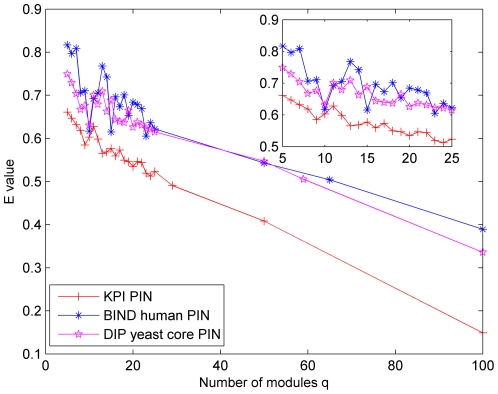
The relationship between E and q on three PINs in the Pinkert method.

**Figure 4:**
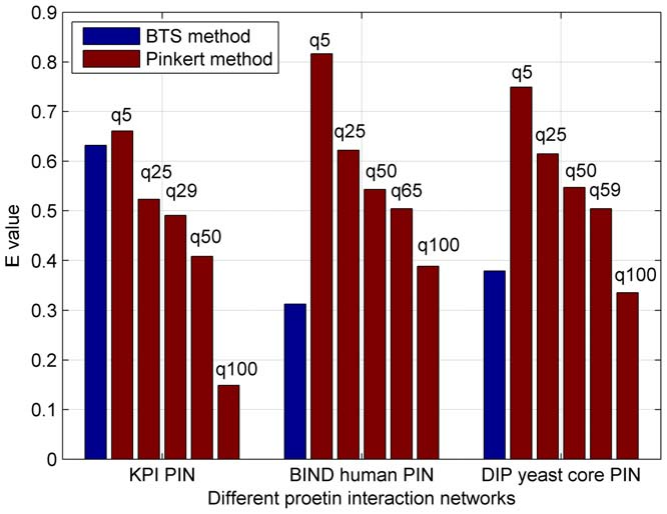
E values comparison results between BTS and Pinkert methods on 3 different PINs. 29, 65, and 59 modules were identified by BTS on the three PINs respectively and results of 5 different number of clusters q of Pinkert method on each PIN were reported.

Different module quality control criterions are available in the literature, for example, the concepts of structural equivalence [31], Newman modularity Q [23], [32], and the E value that describes the connection structure of the original network [20], [33] (see Appendix S2 for definitions of Q and E function). The former two are found as special cases of the E-value used by the Pinkert approach. Therefore, we mainly focus on the comparison of the final E values computed by the proposed BTS method and others. In the Pinkert method, the E-values significantly depend on the predefined number of modules or clusters q and it is still not clear how to determine and select q, which is usually identified by trying different choices. Therefore, in this study, we use the same strategy as in [20] by testing different selections of q, i.e., q = 5 to q = 25, 50, and 100. Figure 3 illustrates the relationship between E and q on the three PINs studied in this paper. From Figure 3, we get an impression that the E values tend to decrease when q increases. In this paper, the typical q = 5, 25, 50 and 100 were selected and their corresponding E values were compared with the BTS method. In addition, the q = 29, 65, and 59 were also set in the Pinkert method on KPI PIN, BIND human PIN, and DIP yeast core PIN respectively because these q values were equal to the outputs from BTS method. The Figure 4 shows the results of comparative E values. As can be seen from Figure 4, the BTS method yields the smaller E values compared with the Pinkert method in BIND human PIN and DIP yeast core PIN (apart from q = 100), which is better according to the definitions of E. In KPI PIN, the E-values by BTS are larger than those generated by Pinkert method in most q selection cases. This could be the existence of some large bi-sparse and cohesive functionally related modules that will be proved by following sections.

**Figure 5:**
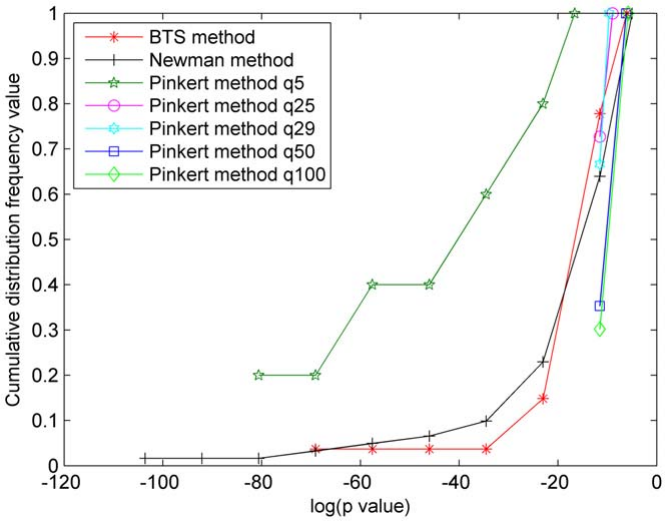
The *p-values* of cumulative distribution frequency of KPIN. The *x*-axis represents log(*p-value*) and the *y*-axis represents the cumulative distribution frequency of the modules of which *p-value* less than the corresponding log(*p-value*) in the *x*-axis.

**Figure 6:**
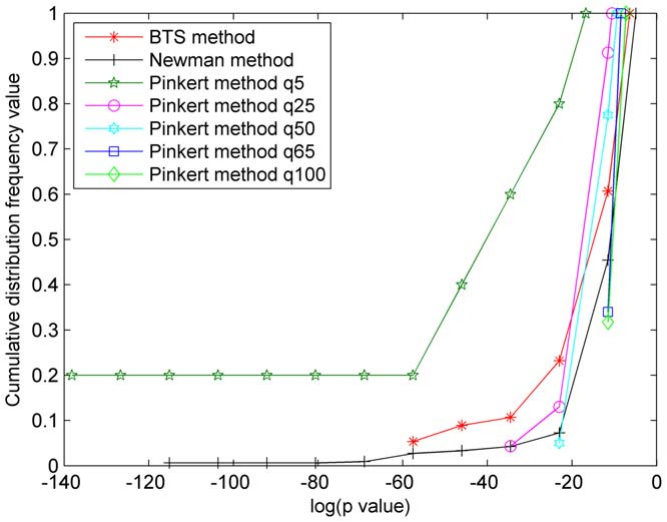
The *p-values* of cumulative distribution frequency of BIND human PIN. The *x*-axis represents log(*p-value*) and the *y*-axis represents the cumulative distribution frequency of the modules of which *p-value* less than the corresponding log(*p-value*) in the *x*-axis.

**Figure 7:**
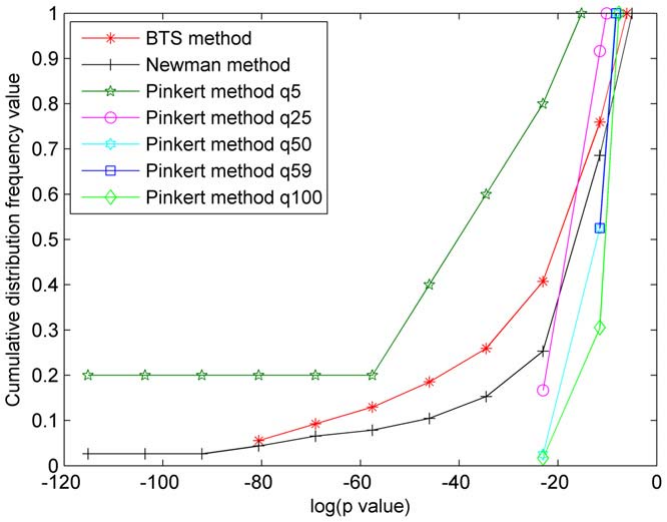
The *p-values* of cumulative distribution frequency of Yeast core PIN. The *x*-axis represents log(*p-value*) and the *y*-axis represents the cumulative distribution frequency of the modules of which *p-value* less than the corresponding log(*p-value*) in the *x*-axis.

In order to evaluate the functional meaningfulness of the obtained modules by the BTS method, Newman-fast method, and the Pinkert method, we performed Gene Ontology (GO) [34] enrichment analysis for all modules using the BiNGO tool [35], which was incorporated into the Cytoscape platform [36]. Based on the BiNGO tool, the number of the modules with no significant annotations and the *p-values* (biological process BP) of all modules are compared. The cumulative distribution frequency of all modules detected by three approaches is employed to explain the results of the *p-values* (see Figure S1 for the detailed results of cumulative distribution frequency and *P-value*s). The performance comparisons are presented in Figures 5, 6, and 7 where it is generally considered to be better if the area under the corresponding curve is larger. As can be seen, the two areas captured by BTS method and Newman method are nearly equal in Figure 5. In Figures 6 and 7, the BTS method achieves the largest area in three methods apart from some cases such as the results generated by the Pinkert method with q = 5.

Then, let's further analyze the cumulative distribution frequency of *P-values* on the microcosmic level. For example, the BTS method captures 5.36% and 12.96% of modules that have at least one enriched GO-term (BP) with *p-value* lower than 10^−25^ in BIND human PIN and yeast core PIN, respectively. Using Newman-fast method, the two values are 2.73% and 7.86%. Unlike the BTS and Newman-fast method, the results of Pinkert method vary significantly with the predefined number of clusters q. As a result, 40% modules have one enriched GO-term with *p-value* lower than 10^−25^ when the q value is set to be 5, and no modules are found to have at least one enriched GO-term with *p-value* lower than 10^−25^ when the q values are set to others on these two PINs. In additional experiment, 3.7% and 4.92% of modules with *p-value* lower than 10^−25^ are obtained when we test the BTS method and Newman-fast method on the KPIN network. Using the same data set, the Pinkert method can identify 40% modules with *p-value* lower than 10^−25^ in a special case (q = 5). These results indicate that the BTS method does capture more effective functional units than the Pinkert method in spite of the low *p-values* obtained by setting small q (q = 5). The outstanding results, which are generated from the Pinkert method with q = 5, is possible because the small q value leads to the formation of large modules, however the meanings of very small number of modules (q = 5) in large PINs are still not clear. In our experiments, we also found that since the Newman-fast method aims to seek maximum Q, it tends to output a few or some large relative dense modules and an amount of small modules especially in sparse networks. Whereas the BTS method tries to simultaneously analyze both bi-sparse and cohesive modules, keep the balances between bi-sparse and cohesive modules by preventing the formation of very large cohesive modules.

**Figure 8:**
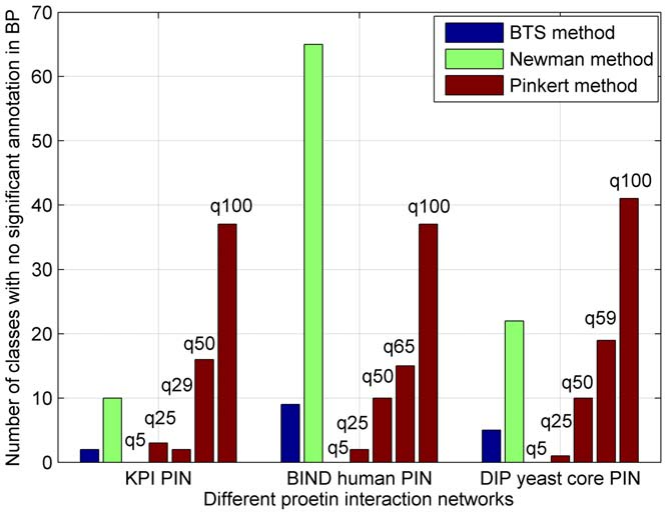
Number of modules with no annotations in BP in Gene Ontology.

**Figure 9:**
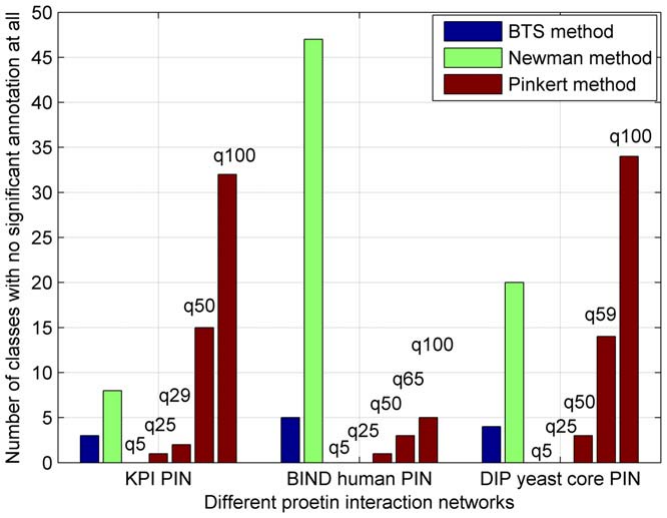
Number of modules with no annotations in all of the three basic categories of Gene Ontology annotations, i.e., biological process, molecular function and cellular component.

Even though a *p-value* gives a good indication about the prominence of a certain functional category, it is risky to draw conclusions solely based on *p-values* [35]. Therefore, we take an additional way that was used in [20] of computing the number of modules that do not have significant enrichment of GO-terms. Figures 8 and 9 show the number of modules which lack enrichment in Biological Process (BP) and in all three basic categories of Gene Ontology annotations, i.e., biological process, molecular function, and cellular component. Since Newman-fast method detects modules (communities) only by optimizing the modularity Q and can not effectively detect modules in sparse networks and get many small modules, the modules of lacking enrichment by Newman-fast method are more than those from the BTS method. As shown in Figure 8, 2/29 module, 9/65 modules, and 5/59 modules obtained by BTS approach lack enrichment in GO BP annotations in KPIN, BIND human PIN, and Yeast core PIN respectively. When evaluating these results with all three categories GO annotations (Figure 9), 3/29 module, 5/65 modules, and 4/59 modules lack enrichment annotations in the tested three PINs. For the Pinkert method, the number of modules without highly significant annotation would increase with the q values become larger. When we set the q value to 5 in the Pinkert method, the number of modules without highly significant annotation in BP or in all three basic categories of GO can be decreased to 0 as shown in Figures 8 and 9. Although most of modules are annotated because there are more proteins in these modules with a small q, these modules lack of significant biological meanings from the statistic *p-values* as shown in Figures 5, 6, and 7. Hence, the Pinkert method would inevitably get into the dilemma when trying to solve the relationship between optimizing the error function value (E) and the number of modules (q) (refer to Figure S1 for the number of modules without highly significant annotations).

### Case studies of mined bi-sparse modules by BTS

**Figure 10:**
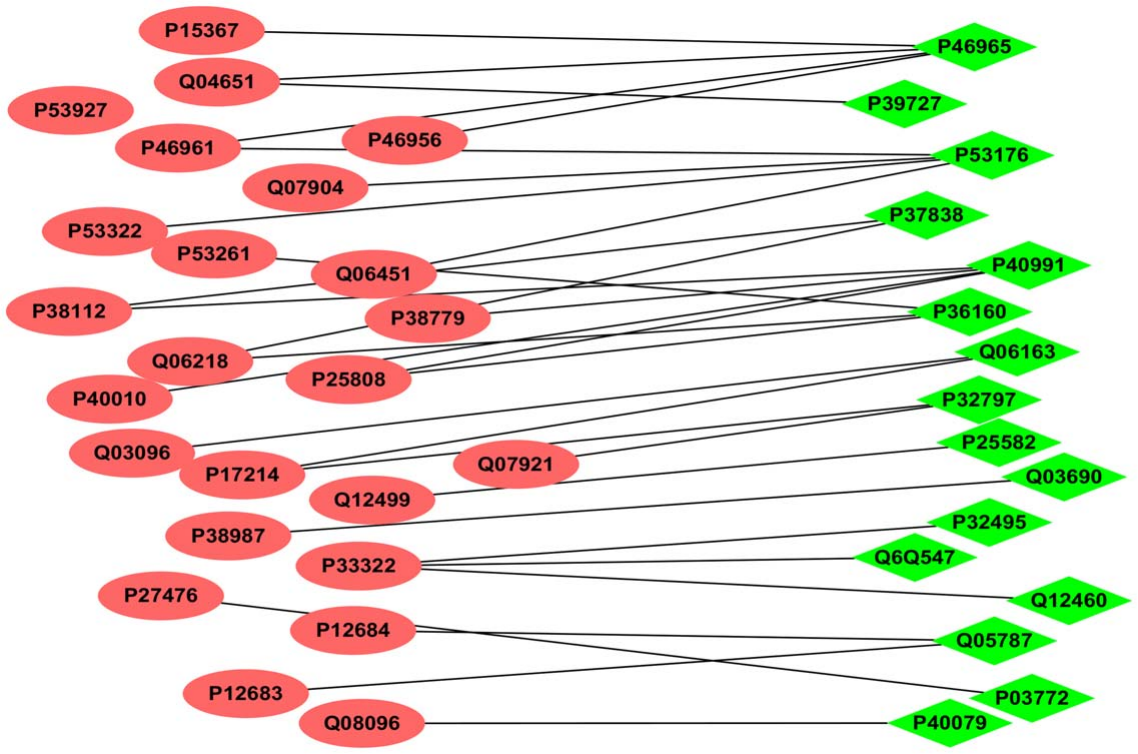
Connections between bi-sparse modules 35 and 36 detected by BTS method in the Yeast core PIN.

In the Yeast core PIN, module 36 is a bi-sparse module with EDM = 0, this cluster is closely related to the biological functions of rRNA modification (*p-value* = 1.7066E-10). Module 36 is found densely connecting with another bi-sparse module 35, whose EDM is also 0 and the members of which are highly enriched under rRNA processing (*p-value* = 1.1915E-8). Figure 10 illustrates the detailed connections between the two bi-sparse modules. This intuitive figure has demonstrated again the importance to develop novel approaches that can effectively find bi-sparse functional clusters in PINs. Apart from Module 36 and 35, bi-sparse module 15 (EDM = 0) containing 261 proteins is also found highly enriched under regulation of biological process (*p-value* = 1.3513E-13) and regulation of cellular process (*p-value* = 2.8968E-13).

In the KPI PIN, module 28 is a bi-sparse module with EDM = 0, which means there are almost no internal links among the 107 nodes in this cluster. However, it is interesting to find that members in module 28 are densely linked with another bi-sparse module 27 in the same network (42 nodes and EDM = 0). Both modules 27 and 28 are significantly enriched in biological regulation (*p-value* = 1.8192E-9) and acetyl-CoA biosynthetic process from pyruvate (*p-value* = 1.8245E-9) respectively. Although the nodes in the two clusters weakly link each other, they do form functional units. It is also found that these two bi-sparse modules strong connect with the cohesive modules 3 and 4 that are involved in nucleolus organization (*p-value* = 2.6173E-7) and protein amino acid phosphorylation (*p-value* = 7.7314E-11) respectively. The other example in KPIN is module 13, which is also a bi-sparse module with EDM = 0, indicating 17 member proteins have no connections with each other. However, they are demonstrated to be closely functional related with protein amino acid phosphorylation (*p-value* = 7.3985E-15) and phosphorylation (*p-value* = 7.4029E-13). By analyzing the distribution results of the above 17 nodes obtained by Newman-fast algorithm, we found 3 members (YGK3, SRP1, CLA4) of were clustered into module 7 in Newman's results, proteins PSK1 and CLB2 were distributed into module12, 2 proteins of YCK1 and SKM1 were fallen into module 11, and the rest 10 proteins were clustered into different modules by Newman-fast algorithm. These results reveal that Newman-fast algorithm prefers to separate the nodes in sparse modules although they are indeed functional related. This bi-sparse module is not found by the Pinkert method either, indicating BTS is more sensitive than the Pinkert method.

In BIND human protein interaction network, module 32 detected by BTS is also a bi-sparse module with EDM = 0. This module contains 167 proteins and 143 of them are protein bindings (*p-value* = 5.175E-40). From the EDM, although all the proteins in the module do not interact with each other, most of them are found to have related functions in signaling (*p-value* = 4.5229E-8) and regulation of catalytic activity (*p-value* = 1.2642E-6).

### Case studies of mined cohesive modules by BTS

In addition, the BTS method can also effectively identify cohesive modules. For example, mined module 3 in KPIN is a cohesive module (EDM = 0.293) that is significantly enriched in protein amino acid phosphorylation (*p-value* = 7.278E-8) and nucleolus organization (*p-value* = 2.6173E-7). Modules 4 (EDM = 0.312), 8 (EDM = 0.216), and 22 (EDM = 0.257) in the Yeast core PIN are the cohesive modules that are involved in transcription (*p-value* = 6.2057E-26), protein import into nucleus (*p-value* = 3.348E-36), and RNA 3′-end processing (*p-value* = 1.5842E-30) functions respectively.

## Discussion

Protein interaction networks are typical complex biological systems that are difficult to be understood from raw experimental data alone. Algorithmic and modeling progresses in the area of biomolecular networks analysis have been demonstrated contributing significantly to the understanding of biological processes and organizations. A common traditional hypothesis is that a functional module in a network is a cohesively linked group of nodes, densely connected internally, and sparsely interacting with the rest of the network. So, many algorithms in the literature try to identify functional modules in PPI networks by searching for such cohesive groups of proteins. However, recent studies have revealed that it is not always the case that members in the functional module link each other densely to form a cohesive cluster. In this paper, a new structure called bi-sparse module was defined, and it would be interesting to answer the question of why bi-sparse subnetworks can compose functional modules. For the bi-sparse modules that link two or multiple bi-sparse or cohesive modules, these proteins might play the role as transport products; for other bi-sparse modules, the homogeneity repulses could be good explanations for the phenomenon.

BinTree Seeking (BTS) method based on the Edge Density of Module (EDM) is proposed to detect both bi-sparse and traditional cohesive modules. Results on three PINs illustrate that BTS can effectively mine functional units, which is better than the approaches that mainly based on maximizing modularity Q (or E), especially in discovering sparse functional clusters. The BTS method also has advantages that can automatically find optimized number of modules in a large PIN. Although BTS method has been demonstrated useful, there is much space to decrease its computational complexity. When applying the current BTS method for analyzing the PIN with more than 10,000 nodes, it could take several days for running depending on the configurations of computation platform. How to speed up BTS and implement a fast algorithm is our future direction. We will also study the effects of different modularity evaluation criteria on the final results in the future. BTS software and the supporting information are available at: www.csbio.sjtu.edu.cn/bioinf/BTS/.

## Materials and Methods

### Materials

In order to verify the universality of bi-sparse functional modules and effectiveness of the proposed BTS method, we applied the BTS approach to three different PINs of different scales. Two experimentally verified yeast protein interaction networks were used. The first one is a global protein Kinase and Phosphatase Interaction Network in yeast (KPIN), which includes 1,844 interactions between 887 protein partners [25]. The other yeast protein interaction network is the DIP “core” set of PPIs and contains 2,147 proteins and 4,275 interactions by removing self-link interactions [27], which is available at http://dip.doe-mbi.ucla.edu/dip/. The third benchmark dataset is human protein interaction network downloaded from the I2D database at: http://ophid.utoronto.ca/ophidv2.201/downloads.jsp. The I2D database [26] is an online database of known and predicted mammalian and eukaryotic protein-protein interactions. It consists of all human protein interaction data sets (including HPRD, BIND, etc). By identifying ‘BIND’ label and removing self-link interactions, we get 3,724 proteins and 8,748 interactions. Detailed information of these 3 PINs is given in the supporting information.

### Methods

The bi-sparse module introduced in this work is a new organizational structure of functional unit in PINs, which is difficult to be mined by applying traditional methods that are specialized for detecting cohesive modules. For example, According to the definition of the modularity Q [16], [23] or E [20], a graph has community structure with respect to a random graph with equal size and expected degree sequence. Therefore, the modularity maximum of a graph reveals a significant community structure only if it is appreciably larger than the modularity maximum of random graphs of the same size and expected degree sequence [37]. Although Reichardt and Bornholdt have studied the issue of the modularity values for random graphs in some depth and proposed the developmental modularity for community detection [38], it was particularly hard to detect communities in sparse graphs by using modularity optimization [37].

**Figure 11:**
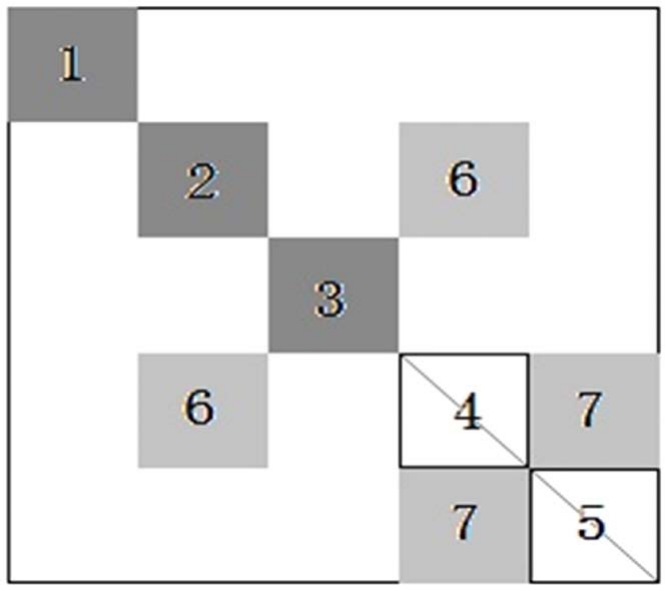
Black blocks (1, 2 and 3), white blocks (4 and 5), and grey blocks (6 and 7) represent cohesive modules, bi-sparse modules and bridge matrixes respectively in an adjacency matrix of a network. Block 6 represents the links between blocks 2 and 4, block 7 represents the links between blocks 4 and 5.

Thus, we propose to achieve this task based on the Edge Density of Module (EDM) and binary tree theory, which is called a BinTree Seeking (BTS). In this new approach, the PIN matrix blocks along the diagonal of the adjacency matrix represent the inner links in the functional modules (both bi-sparse and cohesive modules), and the non-diagonal matrix blocks represent bridge matrix in adjacency matrix of a PIN or links between different functional modules in a PIN. Figure 11 gives an intuitive picture of bi-sparse module, cohesive module, and bridge matrix. Hence, the process of detecting both bi-sparse and cohesive modules is equivalent to optimize the EDMs of the three kinds of modules in an adjacency matrix.

It can be proved that the information of topological interactions in a PIN contained in the matrix is kept unchanged after matrix primary transpositions on the adjacency matrix, such as rearranging the rows and columns, and the information of the rearrangement will be saved in each row and column. We define this quality of adjacency matrix as information synchronization (see Appendix S1 for the proof). Hence, the goal of searching functional modules in a PIN can then be totally achieved by the operations on the adjacent matrix directly. This is different from the conventional approaches in the literature, which detect functional modules by optimizing an objective function of grouping nodes.

Given a module *r*, its EDM is defined as:
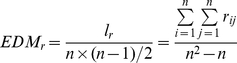
(1)where 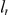 is the actual number of edges in the module *r*, 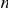 is the total nodes in *r*. We define the Link Density (LD) between a node 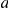 and module 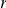 as follows:
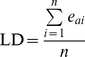
(2)where 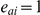 if there is a link between 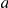 and the *i*-th node in *r*, otherwise 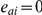.

Edge Density of Bridge Matrix (EDBM) between modules 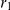 and 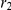, and the Edge Density for a whole Network *R* (or a protein interaction network) (EDN) are defined respectively as:
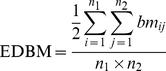
(3)

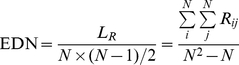
(4)where
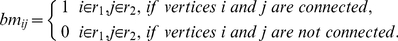
(5)and 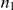, 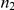 are the number of nodes in modules 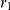 and 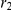 respectively, 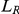 is the actual number of edges a network *R*, 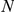 is the total number of nodes in network *R*.

For these definitions, the bridge matrix locates on non-diagonal in adjacency matrix and ensures the links between cohesive modules as few as possible and refines the sizes of bi-sparse modules. Moreover, a priority of operations on adjacency matrix is defined in BTS, i.e., “cohesive module”>“bridge matrix”>“bi-sparse module”. By defining the priority, BTS method can not only successfully capture the functionally cohesive modules defined in traditional approaches because they have the highest priority but also meaningful bi-sparse modules because the priority of “bridge matrix” is higher than “bi-sparse module”, and the “bridge matrix” is capable of refining the sizes of the bi-sparse modules.

**Figure 12:**
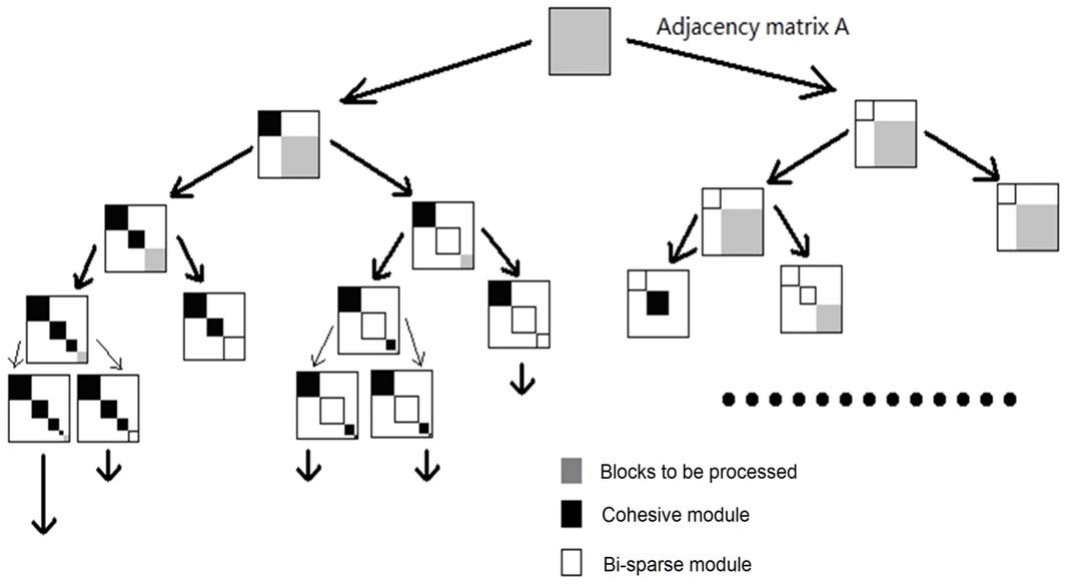
The BinTree Seeking (BTS) method, black blocks, white blocks and grey blocks represent cohesive modules, bi-sparse modules and adjacency matrix blocks to be processed respectively.

**Figure 13:**
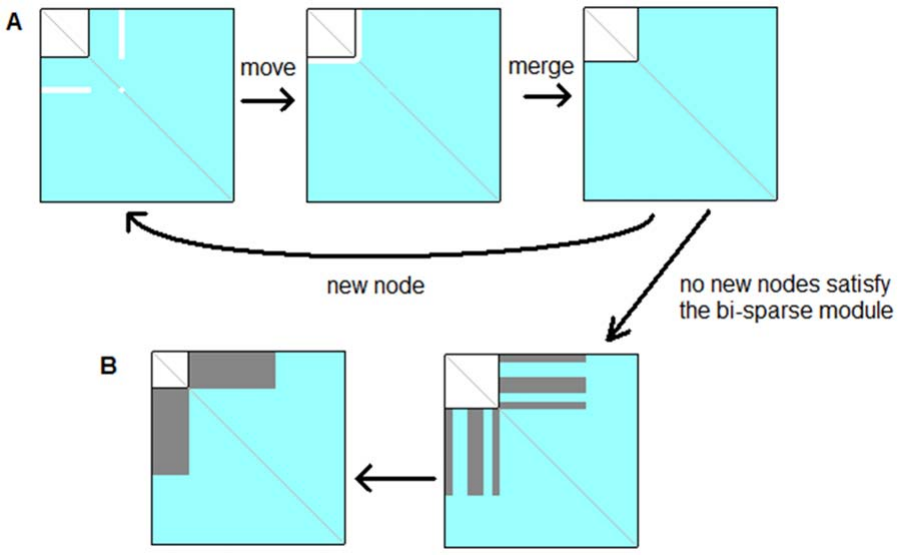
The procedure for building bi-sparse modules, where the white block in adjacency matrix represents bi-sparse modules and the gray blocks represent bridge matrixes. Figure A shows the important steps in constructing the bi-sparse module; and figure B shows how to regulate the bi-sparse module based on its edge density of bridge matrix when some nodes in the bi-sparse module do not meet the threshold *a*_3_.

Depending on the definitions and priority given above, we detect modules from the adjacency matrix of a PIN in following steps. First, by randomly selecting a seed node, we try to build its cohesive modules (left subtree of Figure 12) by adding the nodes whose link density values larger than a threshold *a*_1_ and build its bi-sparse modules (right subtree of Figure 12) by adding the nodes whose link density values smaller than a threshold *a*_2_. As a result, we obtain a Binary Tree (BinTree) with a root (Adjacency matrix) and two leaves, i.e., left subtree represents the adjacency matrix that includes a cohesive modules and the other represents the adjacency matrix that includes a bi-sparse module. Second, the bridge matrixes (block 6 in Figure 11 for example) of the cohesive modules are built if its edge density of bridge matrix value larger than a threshold *a*_3_. Likewise, for the bi-sparse modules obtained by the previous step, its bridge matrixes are also built (block 7 in Figure 11 for example). Third, remove the nodes whose link density values do not meet the threshold *a*_1_ in cohesive module and regulate the bi-sparse module based on its bridge matrixes. Figure 13 gives a simple illustration for building and updating the bi-sparse module. Finally, repeat these steps, until all nodes are processed.

**Figure 14:**
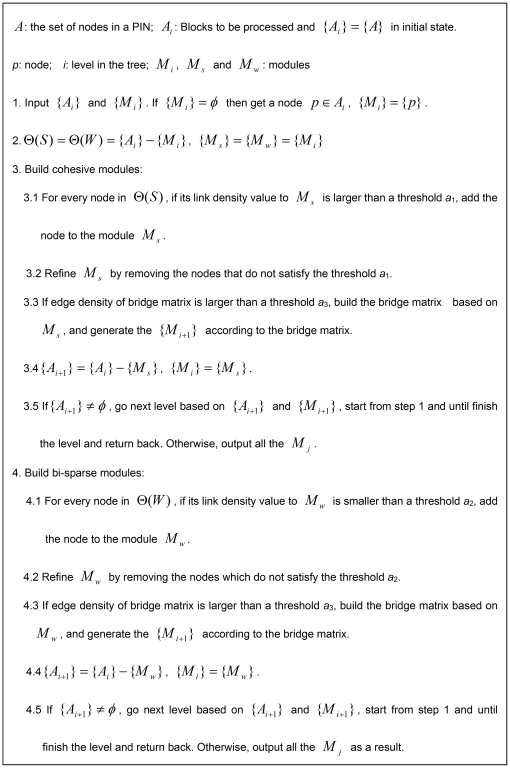
The flowchart for detecting modules from adjacency matrix by BTS.

When all the nodes in the network are classified into different modules, a big bintree is built on the whole network and every pathway in the bintree corresponds to a state to detect modules in a PIN. For all the leave modules in the bintree, we can then evaluate their qualities using some criteria (such as E value) and find the best outputs. The intuitive description of the BTS method and the detailed computation steps are shown in Figures 12 and 14 respectively, where modules on the leftmost path of Figure 12 are similar to modules detected by Newman-fast algorithm [39] that tries to build cohesive modules.

**Figure 15:**
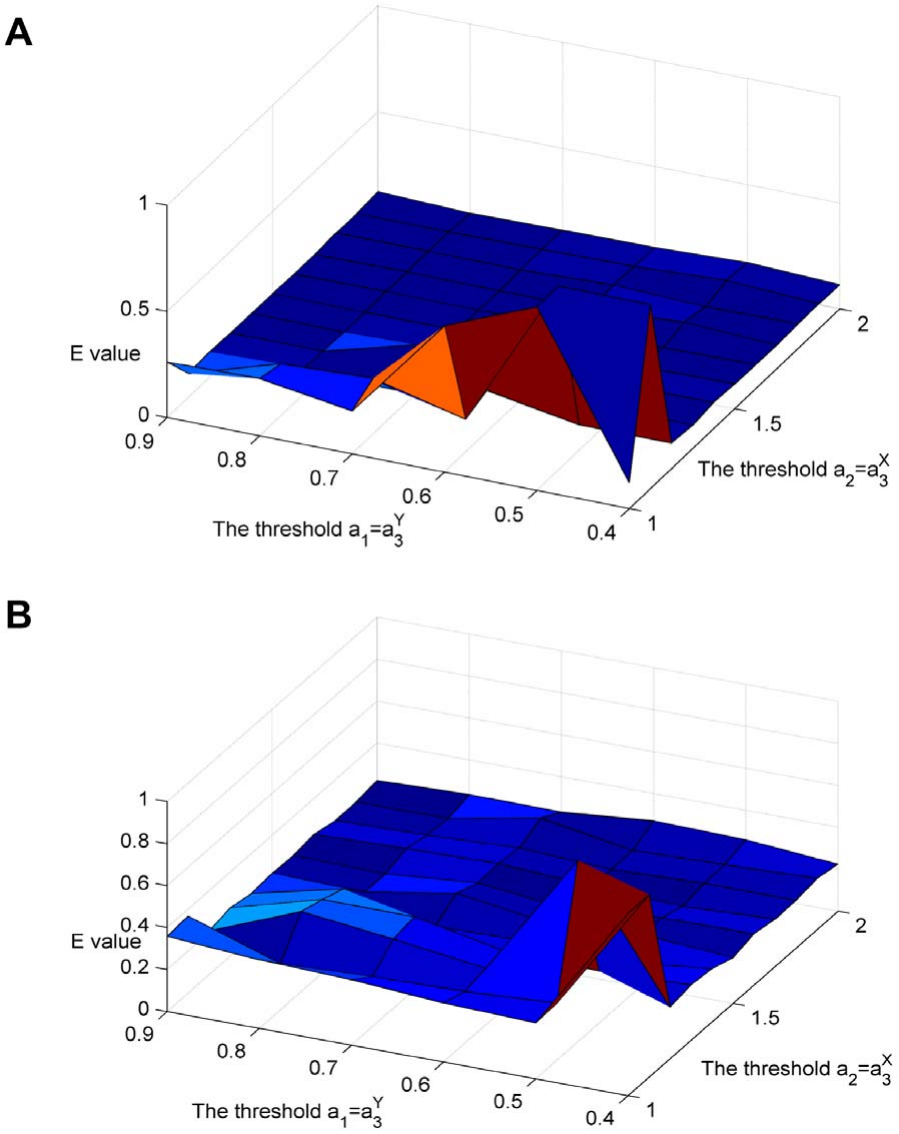
The relationship between the threshold *a_1_*, *a_2_* and E values on the benchmark data with noise level 0 (A) and 0.1 (B) mentioned above, respectively.

As discussed above, the three thresholds (*a*_1_, *a*_2_, *a*_3_) play important roles in BTS. Hence, how to select proper values is a major point. There are some notices for prudently selecting the three thresholds. First, *a*_1_ is the lower limit of the link density of cohesive module; *a*_2_ is the upper limit of link density of bi-sparse module, and *a*_3_ is the lower limit of edge density of bridge matrix required to confirm the existence of bridge matrix. Second, we set *a*_1_<1 and *a*_2_>0, so it can fit the network as a whole to image graphs as good as possible. Third, *a*_1_ and *a*_3_ are the lower limits of the edge density of cohesive matrix blocks and bridge matrixes respectively. So, they should be larger than the upper limit of the edge density of bi-sparse matrix blocks *a*_2_. Due to the priority mentioned above, *a*_1_ is required to be larger than *a*_3_. Therefore, the three threshold values should satisfy *a*_1_>*a*_3_>*a*_2_. According to our experiments, *a*_3_ can be set as the edge density of the given PIN calculated by Eq. (4), and then change the values of *a*_1_ and *a*_2_ accordingly. Figure 15 (A) and (B) illustrate the relationship between *a*_1_, *a*_2_, and the E-values obtained by BTS on the synthetic network. From the two figures, we can see that the combination of 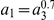 and 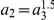 is a significant infection point. E-values tend to increase when 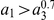 and 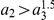, but the various values of *a_1_* and *a_2_* that belong to 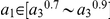 and 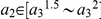 respectively lead to various E values with tiny fluctuation. Although one can get even smaller E values if 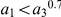 and 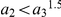, these cases possibly lead to form more small modules. Hence according to the experiments, we select the thresholds as 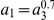 and 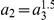 in this study. We also recommend other combinations for these 3 thresholds on the condition that 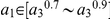 and 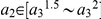.

## Supporting Information

Figure S1 **Detailed outputs from different algorithms on the three real protein-protein interaction networks: module number and members, P-values of modules, and the cumulative distribution frequency values of modules.**
(XLS)

Appendix S1 Matrix primary transpositions on PIN's adjacent matrix keep information synchronization.
(DOC)

Appendix S2 Definitions of Newman Modularity Q and error function E.
(DOC)
