# Supplementary material for: BinTree Seeking: A Novel Approach to Mine Both Bi-Sparse and Cohesive Modules in Protein Interaction Networks
Source: PLoS One. 2011 Nov 28;6(11):e27646. doi: 10.1371/journal.pone.0027646 (PMC3225364; doi:10.1371/journal.pone.0027646)
Supplement: Appendix S1 — Matrix primary transpositions on PIN's adjacent matrix keep information synchronization. (DOC) [file pone.0027646.s002.doc]

**Appendix S1**: Matrix primary transpositions on PIN’s adjacent matrix keep information synchronization.

The information of protein interactions in a PIN contained in the matrix is still complete after rearranging the rows and columns in the same way, and the information of the rearrangement will be saved in each row and column. If we rearrange the rows in the matrix and do the same operations to columns, i.e., exchanging the first row and the second row followed by exchanging the first column and the second column, we can then get a mathematical expression:

$X_{n}=T_{n}X_{0}T_{n}^{'}$ (A1)

where $X_{0}$ is the initial adjacency matrix, $X_{n}$ is the adjacency matrix obtained after a series of exchanges, and $T_{n}$ is defined as:

$T_{n}=\left[\begin{matrix} t_{1} \\ t_{2} \\ \cdots\\ t_{n} \end{matrix}\right],\text{ }t_{j}=[0,0,\cdots,0,1,0,\cdots,0]$ (A2)

Each $t_{j}$ only contains one 1, and $t_{j}$ is not equal to $t_{i}$ ($i\neq j$) in $T_{n}$. We can see that after a series of column exchanges, $T_{n}$ can become an unit matrix $E$:

$\begin{matrix} T_{n}I_{1}I_{2}\cdots I_{n-1}I_{n}=E \\ \Rightarrow T_{n}=I_{n}^{-1}I_{n-1}^{-1}\cdots I_{2}^{-1}I_{1}^{-1}=I_{n}I_{n-1}\cdots I_{2}I_{1} \\ \Rightarrow X_{n}=T_{n}X_{0}T_{n}^{'}=(I_{n}I_{n-1}\cdots I_{2}I_{1})X_{0}(I_{n}I_{n-1}\cdots I_{2}I_{1})^{'} \\ =I_{n}I_{n-1}\cdots I_{2}I_{1}X_{0}I_{1}^{'}I_{2}^{'}\cdots I_{n-1}^{'}I_{n}^{'} \\ =I_{n}I_{n-1}\cdots I_{2}I_{1}X_{0}I_{1}I_{2}\cdots I_{n-1}I_{n} \\ =I_{n}(I_{n-1}\cdots(I_{2}(I_{1}X_{0}I_{1})I_{2})\cdots I_{n-1})I_{n} \\ =I_{n}(I_{n-1}\cdots(I_{3}(I_{2}X_{1}I_{2})I_{3})\cdots I_{n-1})I_{n} \\ =\cdots\\ =I_{n}(I_{n-1}\cdots(I_{i+2}(I_{i+1}X_{i}I_{i+1})I_{i+2})\cdots I_{n-1})I_{n} \end{matrix}$ (A3)

In order to analyze more conveniently, we represent elementary transformation $I_{i+1}$ as row-exchange elementary matrix $I_{kl}$, then the $X_{i+1}=I_{i+1}X_{i}I_{i+1}$ can be written as the form of $X_{i+1}=I_{kl}X_{i}I_{kl}$. The function of this operation is to exchange the information of row *k* and row *l*, column *k* and column *l* as shown in Figure A1.

As the Figure S1_1 shows, the *k*-th and *l*-th rows exchange and the *k*-th and *l*-th matrix units in them also exchange, while in other row vectors, only the *k*-th and *l*-th matrix units exchange. Due to the symmetry property of PIN adjacent matrix, the same changes happen in corresponding column vectors. By analyzing the adjacent matrixes before and after the exchanges, we can discover that the total information of protein interactions have not changed and the only changes are their locations in the adjacency matrix. This means that the final results do not depend on the sequence of the exchanges, i.e., $(I_{kl}X_{i})I_{kl}=I_{kl}(X_{i}I_{kl})$. Thus we can clearly see that the essence of $X_{i+1}=I_{kl}X_{i}I_{kl}$ is to add the exchange information into adjacency matrix, of course, the matrix information will not be lost. Meanwhile, each transformation of $X_{i+1}=I_{i+1}X_{i}I_{i+1}$ has completely kept the adjacency matrix information. Therefore, from the formula $T_{n}=I_{n}I_{n-1}\cdots I_{2}I_{1}$, we can know that the essence of the whole is to add the exchange information into adjacency matrix and all the information of protein interactions is remained in an adjacency matrix of a PIN.


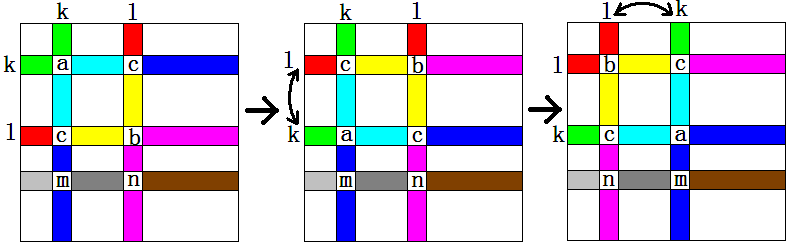


**Figure AS1**. Schematic drawing to show the primary transpositions on PIN’s adjacent matrix.
