# Supplementary material for: BinTree Seeking: A Novel Approach to Mine Both Bi-Sparse and Cohesive Modules in Protein Interaction Networks
Source: PLoS One. 2011 Nov 28;6(11):e27646. doi: 10.1371/journal.pone.0027646 (PMC3225364; doi:10.1371/journal.pone.0027646)
Supplement: Appendix S2 — Definitions of Newman Modularity Q and error function E. (DOC) [file pone.0027646.s003.doc]

**Appendix S2**: Definitions of Newman Modularity Q and error function E.

The most popular quality function is the Modularity Q of Newman and Grivan and can be defined as follows [1]:

$Q=\frac{1}{2m}\sum_{ij} (A_{ij}-P_{ij})\delta(C_{i},C_{j})$ (B1)

where $A$ is the adjacency matrix of the network, $m$ is the total number of edges of the network, and $P_{ij}$ is the expected number of edges between nodes $i$ and $j$ in the null model. The $\delta$ function is one if vertices $i$ and $j$ are in the same module, zero otherwise.

The error function E used in this paper is the same as defined by Pinkert [2]:

$E(\tau,B)=\frac{1}{M}\sum_{i\neq j}^{N} (A_{ij}-B_{\tau_{i}\tau_{j}})(w_{ij}-p_{ij})$ (B2)

where $N$ is the number of nodes in a network, $\tau$ is the mapping of the $N$ nodes to the $q$ different modules. $A_{ij}$ is the adjacency matrix, $w_{ij}$ represents the weight between nodes $i$ and $j$, if an edge is absent in the network, $w_{ij}$ is zero. $B_{\tau_{i}\tau_{j}}$ is the image graph and $p_{ij}$ is a penalty term.

**References**

1. Newman MEJ, Girvan M (2004) Finding and evaluating community structure in networks. Physical Review E 69: 026113.

2. Pinkert S, Schultz J, Reichardt J (2010) Protein Interaction Networks-More Than Mere Modules. Plos Computational Biology 6: e1000659.
